# A Framework to Assess the Citation Performance of Complex Innovation Systems

**DOI:** 10.3389/frma.2021.622006

**Published:** 2021-04-26

**Authors:** Guillermo Armando Ronda-Pupo

**Affiliations:** Departamento de Administración y Economía, Facultad de Ciencias Jurídicas y Empresariales, Universidad de La Frontera, Temuco, Chile

**Keywords:** citation impact analysis, complex innovation system, national science systems, power-law, scale-independent, self-similarity, relative impact

## Abstract

Scientometric indicators are useful to evaluate the relevance of scientific research, to prepare rankings, and to evaluate and inform research policies. That is why the choice of appropriate indicators is a matter of primary concern. This article aims to introduce a framework to decide the appropriate type of indicator for assessing the citation-based performance of complex innovation systems. The framework is two-fold: First, it brings the methodology to decide when the use of standard average based indicators is granted, and when scale-invariant indicators are mandatory. Second, it provides the procedures to build scale-invariant indicators to assess the relative impact of complex innovation systems. The framework is validated empirically through the evaluation of the relative impact of the Chilean science system in 2017. The result suggests that the Chilean science system has characteristics of a complex innovation system such as the distribution of citations fits to a power law with an exponential cutoff −2.77±0.09 and a power-law correlation between the size of the system and its impact 1.29±0.11. Furthermore, the framework shows to be efficient to compare fields of vastly different sizes.

## Introduction

A major feature of frequency distributions of complex innovation systems productivity is their extreme skewness (Glänzel and Nacke, 1988; Braun et al., 1990). Generally, the research system's output such as the number of articles it publishes, the number of patents it registers, or the number of citations it receives, follow approximately a power-law with scaling exponent α≤3.0 (Lotka, 1926; de Solla-Price, 1965; Naranan, 1970; Coile, 1977; Egghe and Rousseau, 1986; Pao, 1986; Glänzel and Nacke, 1988; Naranan, 1989; Narin, 1994; OluicVukovic, 1997; Seglen, 1997; Egghe, 2005; van Raan, 2006; Milojevic, 2010; Bornmann, 2013; Brookes, 2016; Bornmann and Leydesdorff, 2017). These distributions have long tails with exponents in the range between 2 <α≤3 which is the distinctive characteristic of a complex system (Katz, 1999; Katz, 2005; Katz, 2016b; Dorogovtsev and Mendes, 2000; Newman, 2005; Clauset et al., 2009; Castellani and Rajaram, 2016; Rajaram and Castellani, 2016). This quality is the first handicap to overcome when evaluating a system’s research performance using citation-based indicators (de Bellis, 2009; van Raan, 2014a; Katz, 2005; Katz, 1999; Katz, 2016b). As Braun et al. (1990) pointed out, “several attempts have been made to find a suitable method for treating the tail.” It is because a small number of subjects of the population form the tail of the distribution. They do not form a group large enough for any kind of statistical analysis.

The abovementioned handicap comes from the fact that distributions that approximately follow a power-law with exponent in the range 2 <α≤3 have infinite variance (Naranan, 1971; Newman, 2005). It means that they do not belong to the dominion of attraction of Gaussian distributions; hence, the central limit theorem does not apply (Katz and Ronda-Pupo, 2019), and population averages are not appropriate to describe them (Newman, 2011). Likewise, when α≤2, both the mean and the standard deviation are infinite. Katz (2016a) recommended that in these circumstances, the use of scale-invariant indicators will yield equitable indicators.

For any given system, a scale-invariant probability distribution is frequently associated with the system is ruled by self-organization and preferential attachment mechanisms or stated otherwise, that a cumulative advantage process is involved in its behavior (Katz, 2016b; Katz, 2016a). The exponent, also referred to as the scaling factor, of the output of a research system indicates an emergent property of the system, and it can be useful to prepare scale-invariant indicators to characterize it. The determination of the scaling exponent of such distributions is useful that it provides helpful information for decision-making processes with research evaluation purposes. Concretely, it determines when the mean and the standard variation characterize the population correctly, and when it cannot (Katz, 2016b). For example, when the distribution is a power-law with exponents α>3.0. In this situation, one can use the standard indicators based on averages (Katz, 1999).

A second challenge to overcome when building or selecting indicators to assess the research performance of a research system, is the size dependency of citation-based measures (van Raan, 2008; van Raan, 2013; de Bellis, 2009). Regarding this, Martin (2011) posed the provoking question: “How can one come up with an appropriate ‘scale’ to assess and measure the impacts of very different magnitudes?” By way of illustration, how can the scientific community of China be compared with, for example, a country from Latin America? The use of population-based averages, such as citations per article, can produce misleading conclusions because of size-dependent bias. The scaling bias associated with these measures can be eliminated by using a normalization constant assessed through a scaling correlation between citations and articles to more accurately inform policy makers (Katz and Ronda-Pupo, 2019).

Scale-independent indicators are recursive. Any smaller system (field/subfield) contained within the more extensive complex system (domain) will have scale-invariant emergent properties, too (Katz, 2016b). The scale-invariant indicators are useful to compare without bias the research performance of scientific communities of vastly different dimensions, thereby allowing the performance of a small science system to be compared to the performance of a big-sized country or even to the world science system.

Scale invariance is mathematically defined as follows: if p(x) represents a distribution, then $\frac{p\left(x\right)}{p\left(bx\right)}=g\left(b\right)$ for any b (Newman, 2005). It can be understood as follows: If the scale or unit by which x is measured increases by a factor b, then the form of the distribution p(x) stays unaffected, except for a general multiplicative constant (Katz and Ronda-Pupo, 2019). Besides power-law functions, namely those of the form $p\left(x\right)=kx^{\alpha }$, no other mathematical function is scale-invariant.

This study aims to present a framework to assess the citation-based impact of research systems that are characterized by right-skewed distributions that could be described by power-law. The framework uses a two-fold methodology by using the properties of the distributions and the correlation parameters of size and impact of a given research system to decide on what type of indicator, namely scale-independent or average-based, is the appropriate one to be used. The results of such a methodology will lead to results that are unbiased in their formulation, presentation, and research policy information. Furthermore, the values obtained could be useful to compare the performance of the research system under analysis to local, national, regional, or world performances and also to prepare citation-based rankings. All these observations bring us to the following research questions:

*How to build unbiased bibliometric indicators to accurately assess the citation-based performance of complex innovation systems?*
*Is it possible to compare accurately the research performance among complex innovation systems of vastly different sizes?*

## Background

Research evaluation is essentially important to research decision-making processes in research units at all levels, ranging from small research groups to universities and countries (Andras, 2011). The development of a comprehensive and valid research evaluation measure is a crucial precondition for assessing the performance of individual faculty members in academic institutions for promotion and reward purposes (Kirkpatrick and Locke, 1992). Research evaluation encompasses two main distinct groups of stakeholders: first, the government, as the primary research funder, and second, universities, which do the actual research (Johnston and Reeves, 2017).

The assessment of scientific performance within a research system has traditionally been applied through measurements of the number of documents published in peer review journals, for example, those included in the WoS or/and Scopus, by an author affiliated to an institution, a country, a field or a domain and subsequently, the number of citations these articles receive (Pan and Fortunato, 2014). Garfield (2014) persuasively stated, “*Citations have become the currency of scholarship.*” This idea is substantiated by international research evaluation associations quantifying research quality by using citation-based indicators.

Despite the general acceptance of the use of citation-based indicators for research evaluation purposes, the construction of unbiased measures to accurately assess the performance of a research system is an ongoing challenge. The *skewness of citation distributions* and the *size dependence of citations* are among the most challenging issues to accurately build and use unbiased bibliometric indicators to evaluate the performance of a research system (Seglen, 1992; van Raan, 2014b). Attempts to overcome these issues have led to even more complicated and burdensome mechanisms for assessing research performance (Martin, 2011). As Lepori et al. (2011) points out: “Predicting the future performance of research systems has become a difficult assignment which cannot be attended by only financial indicators.”

The scientometric models that are aimed at assessing the impact that articles have on the research community traditionally are built on the number of citations those articles attract. Furthermore, models and indicators reflecting scientific influence on science itself can be classified into two groups namely, 1) traditional models and indicators based on primary publication and citation counts or averages and rankings based on these indicators–an in-depth discussion on these indicators can be found in Waltman (2017) and for its limitations see van Raan (2014a), or 2) models and indicators built on the assumption of the skewed nature of citations counts–for a theoretical and methodological discussions see (Katz, 2005). A unified model that considers both approaches is lacking in the literature. Specifically, it is crucial to have a framework that accurately indicates when to use one or the other of the two approaches mentioned to guarantee that the results are unbiased. A correct choice will ensure that resulting evaluations and policy formulations are not biased.

The framework proposed in this contribution is illustrated by an evaluation of the relative impact of a small science system’s scientific production, namely, Chile in 2017.

## Materials and Methods

The methodology used is two-fold; first, to decide what type of indicators should be used, and second, to assess the relative impact of a domain/field/subfield.

**FIGURE 1:**
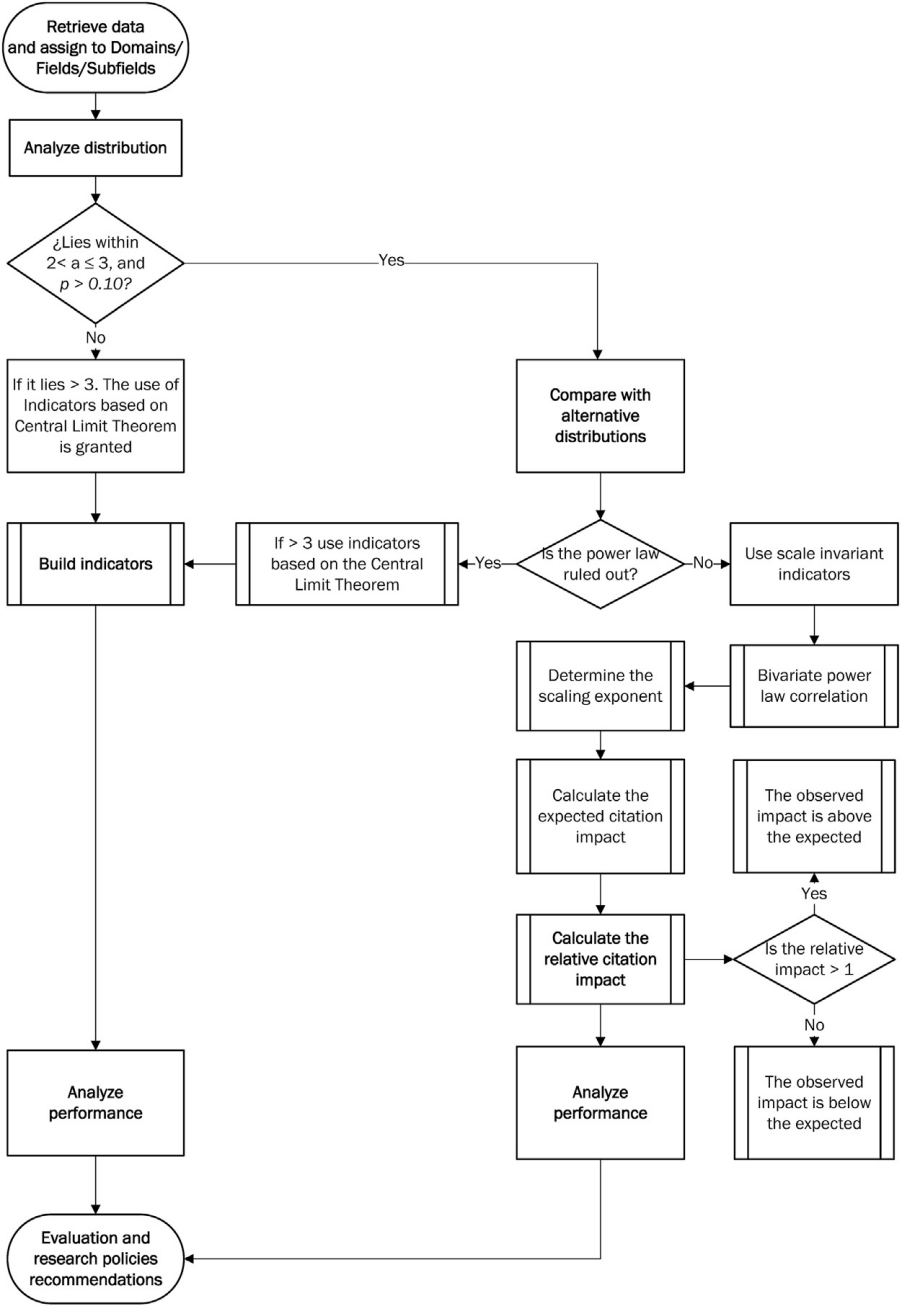
Flowchart of the framework to assess the citation performance of complex innovation systems.

Figure 1 shows the flowchart of the framework. The procedure consists of three main steps with associated tasks depending on specific situations. Each of the steps is explained below.

### First Step: Retrieving and Preparing the Data for Quantitative Analysis

#### The Data Retrieval Strategy

The aim of this step is to retrieve and organize the data for quantitative analysis. The data for the study consist of articles and reviews published by researchers from Chile in the fields of the domains such as Applied Sciences, Economics and Social Sciences, Health Sciences, and Natural Sciences included in the Clarivate Analytics Web of Science™ Core Collection.

#### Assigning Articles to a Unique Field

This step aims to assign each article to a unique domain/field/subfield. The classification of scientific information into an appropriate subject fields is one of the essential preconditions of valid scientometric studies (Haddow, 2015). van Leeuwen and Calero Medina (2012) suggest that the cataloging of publications in the citation indexes with a more in-depth taxonomy scheme would help the assessment of research performance. Herranz and Ruiz-Castillo (2012) stated that about 42% of the documents published in Clarivate Analytics are assigned to between two and up to a maximum of six subfields. This setting generates a drawback in research evaluation using the number of citations. The Science Metrix journal classification ontology overcomes this limitation (Archambault et al., 2015). The Science Metrix journal classification ontology is available under a common creative license. Furthermore, many proficient bibliometricians participated in its formulation. The specific journals are consigned to a unique, mutually exclusive domain/field/subfield using a hybrid procedure conjoining algorithmic techniques and expert judgment (Ronda-Pupo and Katz, 2017). Table 1 presents the research fields studied.

**TABLE 1:** Domains and fields according to *Science Metrix* journal classification.

Domain	Fields
Applied sciences	Agriculture, fisheries, and forestry
	Built environment and design
	Enabling and strategic technologies
	Engineering
	Information and communication technologies
Health sciences	Biomedical research
	Clinical medicine
	Psychology and cognitive sciences
	Public health and health services
Natural sciences	Biology
	Chemistry
	Earth and environmental sciences
	Mathematics and statistics
	Physics and astronomy

Source: Science Metrix, http://science-metrix.com/en/news/science-metrix-launches-the-second-public-release-of-its-multilingual-journal-classification.

### Second Step: Analyzing the Citation Distribution

This step aims to analyze the distribution of citation counts of the publications of the complex innovation system under analysis. Since innovation systems are dynamic and evolve with time, the evolution of a distribution may have to be taken into consideration when determining which functional form is the best fit (Katz 2016). Many computer programs have been created to analyze heavy tail distributions, that is, LOTKA (Rousseau and Rousseau, 2003) and PoweRlaw (Gillespie, 2015; Clauset et al., 2009).

The framework uses Clauset et al. (2009) routine to test the hypothesis of the power-law distribution. The algorithm encompasses three tasks. 1) The assessment of the point where the tail starts$x_{min}$, and the scaling factor or the exponent, 2) Calculate the goodness of fit between the dataset and the fitted distribution, and 3) Compare the power-law with competing distributions. If the power-law is not ruled out and the exponent alpha is inside the range 2 <α≤3, the scale-free measures are required (Katz, 2016a). Conversely, if the distribution has α>3.0 then, one can use either scale-invariant indicators or based on population averages. Both types of indicators would bring unbiased results. The scale-invariant indicators will bring unbiased indicators to compare systems of vastly different sizes.

### Third Step: Preparing the Scale-Invariant Indicators

This step aims to evaluate the relative impact of the domains under analysis. It involves the following tasks: finding the scaling factor of the relationship between size and impact as well as calculating the expected and the relative impact (Ronda-Pupo, 2019). The estimate of the relative impact involves the following tasks: 1) calculating the scaling factor of the relationship between size, and impact, 2) estimating the expected impact, and 3) computing the relative impact.

Below, we describe each step.

#### The Scaling Correlation Between Size and Impact

This step aims to establish the standardization constant. It also looks to find out the exponent of the scaling association between the impact and size. Scaling correlations can occur when entities in a scale-invariant distribution are aggregated into natural groups; for example, when peer-reviewed articles are aggregated into fields (Katz, 2016). Then, a scaling correlation between the impact of a field measured using citations and field size measured using numbers of published articles exists.

The exponent is an extent of the “Matthew Effect,” or the cumulative advantage of citation impact on the size of the system (Katz, 2016b; Ronda-Pupo, 2017).

##### Variables

###### Size

According to Merriam-Webster (2018) dictionary, size is defined as “physical magnitude, extent, or bulk: relative or proportionate dimensions.” The size (S) of a scientific field can be measured, for example, by the number of researchers, the quantity of budget it receives, the number of grants it wins, or the extent of knowledge it produces, among others (Ronda-Pupo, 2017). Frame and Carpenter (1979) initiated analyzing scientific sizes using the number of articles published (see Eq. 1):

$$S_{i}=\overset{j=n}{\underset{j=1}{\sum }}P_{\mathrm{ij}}.$$ (1)

S_i_ is the production of the field i in the journals from $J_{i=1}$ to $J_{n}$ of the field i. That is, the size is the number of articles available in the journals of a field (Ronda-Pupo, 2019). For the present study, the size is the number of articles and reviews published by Chilean researchers in journals of the fields within the domains such as Applied Sciences, Health Sciences, and Natural Sciences in 2017.

###### Impact

Traditionally, the citation impact is expressed as the fraction between the citations and articles (Seglen, 1992). $Citation\;impact=\frac{number\;of\;citations}{number\;of\;papers}$ (Van Raan, 2014a). In the present study, impact (I) is the number of citations to articles published by Chilean researchers in journals of the fields within the domains Applied Sciences, Health Sciences, and Natural Sciences in 2017. Similar to Ronda-Pupo (2019), we used a three-year fixed citation window to calculate the impact of each field. The impact of a field $I_{i}$ is the number of citations received by the articles of the field i in 2017, 2018, and 2019. This procedure guarantees that all documents have equal period probability of getting citations (t3) (see Eq. 2), and prevent bias caused by citation fluctuations:

$$I_{i}=\overset{j=n}{\underset{j=1}{\sum }}I_{\mathrm{ij}}.$$ (2)

##### The Model

The statistical assumptions to run this analysis are as follows: 1) the source population is normally distributed, 2) a constant variance of the dependent variable in the source population, and 3) the independence of residuals. We use Eq. 3 to establish the regression parameters:

I=γ Sα. (3)

Here, I stands for impact, S for size, γ for a standardization constant, and α for the exponent. The logarithmic conversion of Eq. 3 leads to a linear correlation where α, the *exponent*, is specified by

log(I)=α⁡log(S)+log(γ). (4)

The parameters γ and α are calculated using the ordinary least squares because they produce fitted values with the smallest error (Leguendre and Leguendre, 2012) and are also asymmetric (Smith, 2009).

##### The Predictive Ability of the Model

To evaluate the predictive ability of the model, we used the *predicted residual error sum of squares* (PRESS)*.* This statistics is a quantity of how well the power-law model forecasts new data. The smaller the PRESS statistics, the better the predictive power of the model. The PRESS statistics is calculated by summing the squares of the prediction errors.

### Building the Scale-Invariant Indicator

#### The Expected Impact

This step aims to define the expected impact of the systems under analysis, according to its size. The assumption is that the number of citations a research system receives is dependent on its size. The bigger the system is, the more citations it receives. To overcome possible bias in the results, we may answer the question: How many citations are expected the system should receive according to its size? As an example, to answer this question, we substitute S, γ, and α in Eq. 3 with the values in Table 2 (see the Results) to get the expected impact, that is, of Physics and Astronomy field as follows:

$$I_{e}=1.21\;\left(1,453^{1.29}\right)\;\approx 14,524.$$

The expected impact ( $I_{e}$) of the field Physics and Astronomy, giving to its size, is 14,524.

**TABLE 2:** The Chilean science system size and impact, at the field level, in 2017.

Fields	Size	% of overall size	Impact	% of overall impact
Agriculture, fisheries, and forestry	489	7	2278	4
Built environment and design	56	1	357	1
Enabling and strategic technologies	435	6	3524	6
Engineering	445	6	3593	6
Information and communication technologies	171	2	924	1
Biomedical research	539	8	3807	6
Clinical medicine	1243	18	18470	30
Psychology and cognitive sciences	166	2	844	1
Public health and health services	168	2	718	1
Biology	599	9	3395	5
Chemistry	363	5	2110	3
Earth and environmental sciences	417	6	3007	5
Mathematics and statistics	312	5	1080	2
Physics and astronomy	1453	21	17997	29
Overall	6,856	100	62,104	100

Source: Clarivate Analytics Web of Science.

#### The Observed Impact

The observed impact is just the number of citations the articles of the field received in the slice of time analyzed. In the case of Chilean research on Physics and Astronomy the observed impact is 17,997 (see Table 2).

#### The Relative Impact

This step aims to define the relative impact (RI) of each domain using the values of the observed and the expected impact. The relative impact is the proportion of the observed $I_{o}$ and the expected impact $I_{e}$ (Eq. 5) suggested by Katz (2016a). The relative impact is useful to calculate a scale-free indicator specified by

$$\mathrm{RI}\left(x,n\right)=\frac{I_{0}}{I_{e}}.$$ (5)

Following the example of the field Physics and Astronomy, the relative impact is

$$RI=\;\frac{17,997}{14,524}\;\approx 1.24.$$

### Using the Scale-Invariant Indicator to Evaluate the Citation Performance

The objective of this phase is to evaluate what the impact of the domain is according to its size. The interpretation of the results is as follows:

RI=1.0.

If the relative impact *RI* is equal to one, there is not a cumulative advantage of the system on its size:

RI >1.0.

If the value of the relative impact is higher than one, the observed impact is over what is expected. The domain is displaying a cumulative advantage as its size increases:

RI<1.0.

Conversely, if the relative impact is less than the unity, the observed impact is under what it is estimated to be according to its size. The system is not returning much impact as expected, giving to its size. The system is displaying a cumulative disadvantage or negative Matthew Effect as its size increases (Katz and Cothey, 2006).

If one uses this indicator to prepare ranks and/or to compare research assessment among systems of dissimilar sizes, the one with the greater relative impact, RI, will be considered the field with the highest impact.

The value RI>1.0 of the field Physics and Astronomy calculated in the preceding step implies that the observed impact of this research field is above the expected according to its size (scientific production). There is a positive return, cumulative advantage or Mattew Effect of its impact on the growth of its size. Below, we use the framework to estimate the citation performance of the Chilean science system in 2017.

## Results

### The Citation-Based Performance of the Chilean Science System in 2017

#### First Step: The Data

The data for the experiment consists of 6,856 articles and reviews published by Chilean researchers in the Clarivate Analytics Web of Science database in 2017 that received 62,104 citations, considering a fixed three-year citation window. We include only the documents published in the fields of the domains such as Applied Sciences, Health Sciences, and Natural Sciences, using the Science Metrix journal classification schema.

Table 2 presents the size and the impact of each field. Four fields (39%) accounted for 54% of the overall productivity, and 69% of the overall impact of the Chilean science system.

### Second Step: The Analysis of the Distribution

**FIGURE 2:**
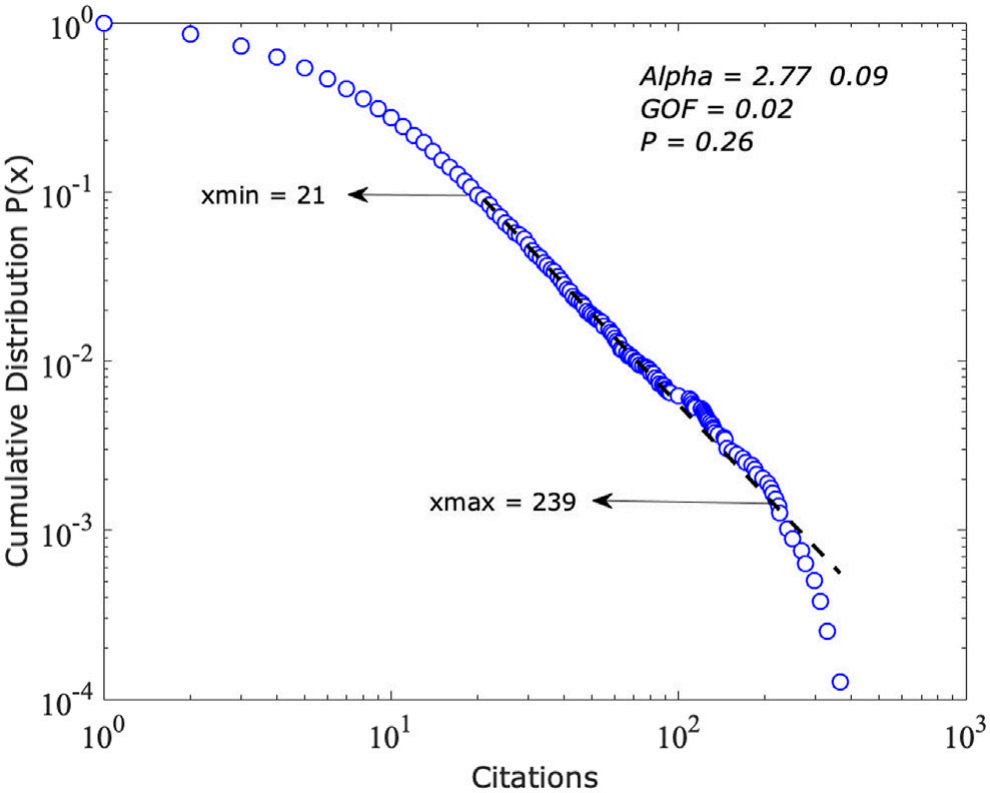
The cumulative distribution function of citations of the power-law model; x = citations.

The exponent of the distribution of the citations is −2.77±0.09 (see Figure 2; Table 3). This is consistent with (Katz, 2016) suggestion that the scaling exponents for distributions of smaller populations within the aggregate population may have exponents ≤3.0. According to Clauset et al. (2009) procedures, the p-value is significant. Next, the power-law distribution will be compared to alternatives.

**TABLE 3:** Results of fitting the power law to the citation distribution.

Dataset	$x_{\min}$	α	*p*	*KS*
Citations 2017	21 ± 4.46	−2.77 ± 0.09	0.26	0.02

*KS* is the Kolmogorov–Smirnov test.

Table 4 presents the outcomes of comparing the power-law to competing distributions. The power-law distribution is ruled out by the power-law with cutoff −11.96, P=0.00. This result confirms Katz (2016) that in the early stages the distribution may be exponential or lognormal evolving into a power-law with an exponential cutoff and eventually become a pure power-law. For this characteristic, indicators based on population averages are not accurate to characterize or to evaluate the citation performance of this research system (Braun et al., 1990; van Raan, 2014a; Katz, 2016a). The use of scale-adjusted indicators will bring unbiased results.

**TABLE 4:** Results of the comparison of the power law with alternative distributions.

Dataset	*p*	Poisson		Lognormal		Exponential		Stretched exponential		Power law + cutoff		Support for power law
		*LR*	*p*	*LR*	*p*	*LR*	*p*	*LR*	*p*	*LR*	*p*	
Chile 2017	0.26	7.40	0.00	−0.47	0.63	5.91	0.00	5.25	0.00	−11.96	**0.00**	Power law + cutoff

*LR* is the log likelihood ratio test. Bold value is the distribution that best fit the data.

### Third Step: Preparing the Scale-Invariant Indicators

#### The Correlation Between Impact and Size

**FIGURE 3:**
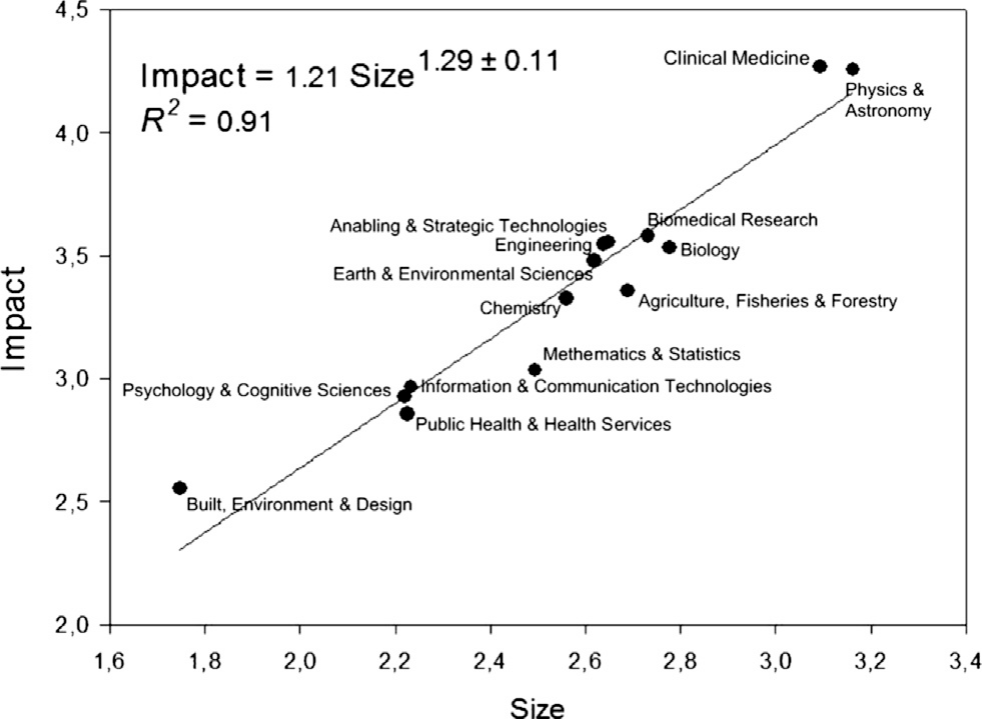
Power-law correlation of impact on size.

The population normality around the regression line (Shapiro−Wilk, P=0.99), the constant variance of the dependent variable (P=0.58), and independence of residuals (Durbin−Watson = 2.16) were tested and met. Figure 3 shows the exponent for the scaling relationship between impact and size. The impact grows nonlinearly with the size of the field $2^{1.29}$ or 2.44  times when the size of a field doubled. The exponent >1.0 indicates there is a super linear correlation between impact and size and a cumulative advantage of impact as the size of the system increases. The correlation is statistically significant $t\left(1,\;13\right)=11.66,\;R^{2}=0.91,\;P=0.001$. The value 0.39 of the *PRESS* statistics supports the accurateness of the model.

#### The Relative Impact

Table 5 shows that the relative impact of the fields Built Environment and Design, Biomedical Enabling and Strategic Technologies, Engineering, Information and Communication Technologies, Clinical Medicine, Earth and Environmental Sciences, and Physics and Astronomy is above the expected according to their sizes (RI>1.00).

**TABLE 5:** The observed, expected, and relative impact of the Chilean science system.

Fields	Size	OI	EI	RI	Performance
Agriculture, fisheries, and forestry	489	2278	3564	0.64	<
Built environment and design	56	357	218	1.64	>
Enabling and strategic technologies	435	3524	3065	1.15	>
Engineering	445	3593	3156	1.14	>
Information and communication technologies	171	924	919	1.01	>
Biomedical research	539	3807	4041	0.94	<
Clinical medicine	1243	18470	11876	1.56	>
Psychology and cognitive sciences	166	844	885	0.95	<
Public health and health services	168	718	898	0.80	<
Biology	599	3395	4631	0.73	<
Chemistry	363	2110	2427	0.87	<
Earth and environmental sciences	417	3007	2902	1.04	>
Mathematics and statistics	312	1080	1996	0.54	<
Physics and astronomy	1453	17997	14525	1.24	>

The symbol > indicates that the observed impact is above the expected.

OI, observed impact; EI, expected impact; RI, relative impact.

Built Environment and Design show the highest relative impact. The impact of this field shows a high citation-based performance according to its size. This field is ranked first with an observed impact about 22 times less than the field ranked second, Clinical Medicine. This field would be placed in the seventh place using standard average impact measures. The results suggest that the use of size independent measures is a correct choice to evaluate the citation-based performance of scientific fields, and to compare or prepare rankings of research systems of pronounced differences in size.

## Discussion and Final Remarks

Chile is placed fourth in Latin America according to its scientific production in the Web of Science. Katz (2016b) claimed: “The global research system has the general characteristics of a complex system,” and then pose the hypothesis if it can be revealed that a property is scale-invariant at higher levels of aggregation; then it can be assumed with increased conviction that it is expected to be scale-invariant at low levels too. The results support the Katz (2016b) suggestion. The Chilean science system as a scaled level of the world science system is a complex innovation system too. It is characterized by scale-invariant properties such as the following.

### The Distribution of Citations Follows a Power Law With Exponential Cutoff

A power-law with an exponential cutoff (−11.96, P=0.001) fits better than the distribution of citations with an exponent α≈−2.77 ±0.09. This exponent α<3.0 is consistent with Katz (2016b) findings that for some subfields the exponents became <3.0 within the first few years of their evolution. The exponent α<3.0 denotes that using the scale-free indicators is the correct choice to assess accurately the citation-based performance of the system. This result contributes to provide a solution to the drawback that the skewness of citations distributions pose to research evaluation purposes as it has been systematically highlighted by van Raan (2014a), Katz (1999), Braun et al. (1990), Egghe and Rousseau (1986). Furthermore, this result confirms Katz (2016b) conclusions that the distribution of impact and the correlation between impact and size at points in time have scale-invariant properties. This result answers the first research question posed in the study.

As a practical implication, the result brings the empirical evidence to the Chilean policy makers to correctly decide the appropriate quantitative indicators to evaluate the Chilean innovation system’s citation impact.

### The Scaling Correlation Between Size and Impact

The relationship between impact and size show a scaling relationship according to a power-law with an exponent 1.29±0.11. Katz (2016b), Table 6 reports a similar scaling exponent for MAPS. The scaling exponent is also similar to the one found by van Raan (2020) for the scaling of the gross urban product for all (kreisfreize) cities and Kreize in Germany. The parameters of the power-law correlation are useful to prepare scale-independent indicators solving the equation $Impact=1.21\;Size^{1.29}$ for each field. Katz (2016b) states that this scale-invariant association can be used as an indication function to determine a scale-independent measure of how much impact a field is having relative to the average system impact. This result contributes to accounting for the size dependence of citation impact, which has been alerted by Katz and Cothey (2006), Katz (2005), Katz (2000), van Raan (2013), Martin (2011). This measure is also useful to compare and rank complex innovation systems of vastly different sizes.

**TABLE 6:** Ranking using diverse scientometric indicators.

Fields	Relative impact	Citations/productivity	Productivity	Citations
Built environment and design	1	7	14	14
Clinical medicine	2	1	2	1
Physics and astronomy	3	2	1	2
Enabling and strategic technologies	4	3	7	5
Engineering	5	4	6	4
Earth and environmental sciences	6	5	8	7
Information and communication technologies	7	10	11	11
Psychology and cognitive sciences	8	11	13	12
Biomedical research	9	6	4	3
Chemistry	10	8	9	9
Public health and health services	11	13	12	13
Biology	12	9	3	6
Agriculture, fisheries, and forestry	13	12	5	8
Mathematics and statistics	14	14	10	10

Fields are ranked according to the relative impact.

The resulting scaling exponent 1.29 describes the Chilean innovation system citation network’s self-similar property composed of its research fields. This exponent is constant irrespective of the field’s size. This scaling correlation also suggests that the average scientific impact, which is commonly used by Chilean research evaluation institutions, is not normalized for field size. If one divides both sides of the scaling correlation by P, field size, the resulting equation has a scaling exponent much less than if C/P were normalized. It should be constant for changing size. The results confirm van Raan (2008) asseveration that citation networks’ scaling correlation appears to be the rule, not the exception.

### Scale-Independent Measures and Policy Evaluation

The scale-independent measure prepared is useful to evaluate the research fields’ relative impact. Table 6 shows rankings prepared using different scientometric indicators. Built Environment and Design, is placed last in the ranking according to its productivity, last according to the number of citations, seven according to its average citations is placed first if the ranking is prepared using the scale-invariant indicator, namely the relative impact. This result reaffirms the effectiveness of the scale-free indicators to prepare rankings, and to compare the performance among fields of vastly different sizes. It is possible to compare the impact of a research field, namely Built Environment and Design to another that is 22 times bigger in size, and achieve a better performance. This result shows the efficacy of the scale-invariant functions used to create the scale-free model used. These measures ensure that policy makers get a reliable evidence-based view of the innovation systems that are the focus of their policies. This result answers the second research question of the study.

The Chilean science system is a young small research system whose citation network shows scale-invariant properties. The result suggests the Chilean policy-making agencies as Agencia Nacional de Investigación y Desarrollo (ANID) and funding institutions as Fondo Nacional de Desarrollo Científico y Tecnológico (FONDECYT) should pay special attention to the scale-invariant properties of the Chilean innovation system with research assessment purposes. The use of standard average evaluation indicators like citations per article would bring biased results. The formulation or information of public research policies based on those results will be misleading. Furthermore, the results suggest that the Chilean research evaluation policy should use scale-invariant indicators and enhance a mix of quantitative and qualitative indicators to bring a more insightful evidence-based research quality evaluation and avoid the overuse of journals’ impacts on the research assessment processes.

Future research is advised to perform thorough comparisons on the effect that field-normalized and scale-adjusted measures have on the rankings of performance measures from distributions with scaling exponents ≤3.0 or with a mixture of scaling exponents ≤3.0 and>3.0 as suggested by Katz (2016b).

## Data Availability

The datasets presented in this article are not readily available because the author confirms that, for approved reasons, some access restrictions apply to the data underlying the findings. The data have been obtained from Clarivate Analytics' Web of Science through the Chilean National Foundation for Science and Technology (FONDECYT) agreement with Clarivate Analytics who do not allow making the data freely available. Requests to access the datasets should be directed to Clarivate Analytics Web of Science.

## References

[B1] Andras P. (2011). Research: metrics, quality, and management implications. Res. Eval. 20 (2), 90–106. 10.3152/095820211x12941371876265

[B2] Archambault É. Beauchesne O. H. Caruso J. (2015). Towards a multilingual, comprehensive and open scientific journal ontology. Available at: http://www.science-metrix.com/pdf/Towards_a_Multilingual_Comprehensive_and_Open.pdf (Accessed March 30, 2016).

[B3] Bornmann L. (2013). How to analyze percentile citation impact data meaningfully in bibliometrics: the statistical analysis of distributions, percentile rank classes, and top-cited papers. J. Am. Soc. Inf. Sci. Tec 64 (3), 587–595. 10.1002/asi.22792

[B4] Bornmann L. Leydesdorff L. (2017). Skewness of citation impact data and covariates of citation distributions: a large-scale empirical analysis based on web of science data. J. Informetr. 11 (1), 164–175. 10.1016/j.joi.2016.12.001

[B5] Braun T. Glänzel W. Schubert A. (1990). Publication productivity: from frequency distributions to scientometric indicators. J. Inf. Sci. 16, 37–44. 10.1177/016555159001600107

[B6] Brookes B. C. (2016). “Sources of information on specific subjects” by S.C. Bradford. J. Inf. Sci. 10 (4), 173–175. 10.1177/016555158501000406

[B7] Castellani B. Rajaram R. (2016). Past the power law: complex systems and the limiting law of restricted diversity. Complexity 21 (S2), 99–112. 10.1002/cplx.21786

[B8] Clauset A. Shalizi C. R. Newman M. E. J. (2009). Power-law distributions in empirical data. SIAM Rev. 51 (4), 661–703. 10.1137/070710111

[B9] Coile R. C. (1977). Lotka's frequency distribution of scientific productivity. J. Am. Soc. Inf. Sci. 28 (6), 366–370. 10.1002/asi.4630280610

[B10] de Bellis N. (2009). Bibometrics and citation analysis: fron the science citation index to cibermetrics. Toronto: The Scarecrow Press, Inc.

[B11] de Solla Price D. J. (1965). Networks of scientific papers. Science 149 (3683), 510–515. 10.1126/science.149.3683.510 14325149

[B12] Dorogovtsev S. N. Mendes J. F. F. (2000). Scaling behaviour of developing and decaying networks. Europhys. Lett. 52 (1), 33–39. 10.1209/epl/i2000-00400-0

[B13] Egghe L. (2005). Powerlaws in the information production process: Lotkaian informetrics. Uk: Elsevier Academic Press.

[B14] Egghe L. Rousseau R. (1986). A characterization of distributions which satisfy Price's Law and consequences for the Laws of Zipf and Mandelbrot. J. Inf. Sci. 12 (4), 193–197. 10.1177/016555158601200406

[B15] Frame J. D. Carpenter M. P. (1979). Int. Res. Collaboration. Soc. Stud. Sci. 2, 481–497. 10.1177/030631277900900405

[B16] Garfield E. (2014). 50 years of citation indexing*:* a visit with Dr. Eugene Garfield. Manhattan, NY: Thomsom Reuters Available at: https://www.youtube.com/watch?v=2kZ0_5HTYDQ (Accessed August 27, 2020).

[B17] Gillespie C. S. (2015). Fitting heavy tailed distributions: the poweRlaw package. J. Stat. Softw. 64 (2), 1–16. 10.18637/jss.v064.i02

[B18] Glänzel W. Nacke O. (1988). “Distributions in bibliometrics: significance and analysis,” in Proceedings of the “Deustscher Documentartag”, Aachen. Chennai: FRG, 88.

[B59] Haddow G. (2015). Research classification and the social sciences and humanities in Australia: (Mis)Matching organizational unit contribution and the impact of collaboration. Res. Eval. 24, 325–339. 10.1093/reseval/rvv006

[B19] Herranz N. Ruiz-Castillo J. (2012). Sub-field normalization in the multiplicative case: high- and low-impact citation indicators. Res. Eval. 21 (2), 113–125. 10.1093/reseval/rvs006

[B20] Johnston J. Reeves A. (2017). Assessing research performance in UK universities using the case of the economics and econometrics unit of assessment in the 1992–2014 research evaluation exercises. Res. Eval. 26, rvw021. 10.1093/reseval/rvw021

[B21] Katz J. S. Cothey V. (2006). Web indicators for complex innovation systems. Res. Eval*.* 15 (2), 85–95. 10.3152/147154406781775922

[B22] Katz J. S. (1999). The self-similar science system. Res. Pol. 28 (5), 501–517. 10.1016/S0048-7333(99)00010-4

[B23] Katz J. S. (2000). Scale-independent indicators and research evaluation. Sci. Public Pol. 27 (1), 23–36. 10.3152/147154300781782156

[B24] Katz J. S. (2005). Scale-independent bibliometric indicators. Meas. Interdiscip. Res. Perspective 3 (1), 24–28. 10.1207/s15366359mea0301_3

[B25] Katz J. S. (2016a). “Policies considerations for evidence-based measures of complex innovation systems,” in SPRU 50th Aniversary Conference, September 5-9, 2016. Sussex, United Kingdom: University of Sussex.

[B26] Katz J. S. (2016b). What is a complex innovation system? PLos One 11 (6), e0156150e0156150. 10.1371/journal.pone.0156150 27258040 PMC4892634

[B61] Katz J. S. Ronda-Pupo G. A. (2019). Cooperation, scale-invariance and complex innovation systems: a generalization. Scientometrics 121, 1045–1065. 10.1007/s11192-019-03215-8

[B27] Kirkpatrick S. A. Locke E. A. (1992). The development of measures of faculty scholarship. Group Organ. Manage. 17 (1), 5–23. 10.1177/1059601192171002

[B28] Leguendre P. Leguendre L. (2012). “Numerical ecology,” in Developments in environmental modelong. 3 ed. (Great Britain: Elsevier B. V), Vol. 24.

[B29] Lepori B. Reale E. Tijssen R. (2011). Designing indicators for policy decisions: challenges, tensions and good practices: introduction to a special issue. Res. Eval. 20 (1), 3–5. 10.3152/095820211x12941371876229

[B30] Lotka A. J. (1926). The frequency distribution of scientific productivity. J. Wash. Acad. Sci. 16 (12), 317–323. Available at: http://www.jstor.org/stable/i24527553.

[B31] Martin B. R. (2011). The Research Excellence Framework and the 'impact agenda': are we creating a Frankenstein monster? Res. Eval. 20 (3), 247–254. 10.3152/095820211x13118583635693

[B32] Merriam-Webster (2018). Merriam Webster dictionary. Available at: https://www.merriam-webster.com/dictionary (Accessed August 26, 2020).

[B33] Milojevic S. (2010). Power law distributions in information science: making the case for logarithmic binning. J. Am. Soc. Info. Sci. Tech. 61 (12), 2417–2425. 10.1002/asi.21426

[B34] Naranan S. (1970). Bradford's law of bibliography of science: an interpretation. Nature 227 (5258), 631–632. 10.1038/227631a0 5429302

[B35] Naranan S. (1971). Power law relations in science bibliography-a self‐consistent interpretation. J. Documentation 27 (2), 83–97. 10.1108/eb026510

[B36] Naranan S. (1989). “Power law” version of Bradford's law: statistical tests and methods of estimation. Scientometrics 17 (3-4):211–226. 10.1007/Bf02026411

[B37] Narin F. (1994). Patent bibliometrics. Scientometrics 30 (1), 147–155. 10.1007/BF02017219

[B38] Newman M. (2005). Power laws, Pareto distributions and Zipf's law. Contemp. Phys. 46 (5), 323–351. 10.1080/00107510500052444

[B39] Newman M. E. J. (2011). SIGMETRICS posting. Available at: http://mail.asis.org/mailman/private/sigmetrics/2011-September/005797.html (Accessed August 10, 2020).

[B40] OluicVukovic V. (1997). Bradford’s distribution: from the classical bibliometric ‘‘Law’’ to the more general stochastic models. J. Am. Soc. Info. Sci. Tech. 48 (9), 833–842. 10.1002/(SICI)1097-4571(199709)48:9<833::AID-ASI7>3.0.CO;2-S

[B41] Pan R. K. Fortunato S. (2014). Author Impact Factor: tracking the dynamics of individual scientific impact. Sci. Rep. 4, 4880. 10.1038/srep04880 24814674 PMC4017244

[B42] Pao M. L. (1986). An empirical examination of Lotka's law. J. Am. Soc. Inf. Sci. 37 (1), 26–33. 10.1002/asi.463037010510.1002/(sici)1097-4571(198601)37:1<26::aid-asi4>3.0.co;2-z

[B43] Rajaram R. Castellani B. (2016). An entropy based measure for comparing distributions of complexity. Phys. A: Stat. Mech. its Appl. 453, 35–43. 10.1016/j.physa.2016.02.007

[B44] Ronda-Pupo G. A. Katz J. S. (2017). The scaling relationship between citation-based performance and coauthorship patterns in natural sciences. J. Assoc. Inf. Sci. Technol. 68 (5), 1257–1265. 10.1002/asi.23759

[B45] Ronda-Pupo G. A. (2017). The citation-based impact of complex innovation systems scales with the size of the system. Scientometrics 112 (1), 141–151. 10.1007/s11192-017-2401-3

[B60] Ronda-Pupo G. A. (2019). The performance of Latin American research on economics & business. Scientometrics 122, 573–590. 10.1007/s11192-019-03300-y

[B46] Rousseau B. Rousseau R. (2003). LOTKA: a program to fit a power law distribution to observed frequency data. Int. J. Scientometrics, Informetrics Bibliometrics 4 (1), 1–6.

[B48] Seglen P. O. (1992). The skewness of science. J. Am. Soc. Inf. Sci. 43 (9), 628–638. 10.1002/(sici)1097-4571(199210)43:9<628::aid-asi5>3.0.co;2-0

[B49] Seglen P. O. (1997). Why the impact factor of journals should not be used for evaluating research. Br. Med. J. 314, 498–502. 10.1136/bmj.314.7079.497 9056804 PMC2126010

[B50] Smith R. J. (2009). Use and misuse of the reduced major axis for line-fitting. Am. J. Phys. Anthropol. 140 (3), 476–486. 10.1002/ajpa.21090 19425097

[B51] van Leeuwen T. N. Calero Medina C. (2012). Redefining the field of economics: improving field normalization for the application of bibliometric techniques in the field of economics. Res. Eval. 21 (1), 61–70. 10.1093/reseval/rvr006

[B52] van Raan A. F. J. (2006). Statistical properties of bibliometric indicators: research group indicator distributions and correlations. J. Am. Soc. Inf. Sci. 57 (3), 408–430. 10.1002/asi.20284

[B53] van Raan A. F. J. (2008). Scaling rules in the science system: influence of field-specific citation characteristics on the impact of research groups. J. Am. Soc. Inf. Sci. 59 (4), 565–576. 10.1002/asi.20765

[B54] van Raan A. F. J. (2013). Universities scale like cities. PLoS One 8 (3), e59384e59384. 10.1371/journal.pone.0059384 23544062 PMC3609770

[B55] van Raan A. F. J. (2014a). Advances in bibliometric analysis: research performance assessment and science mapping. Dordretch: Portland Press.

[B56] van Raan A. F. J. (2014b). “Bibliometrics: use and abuse in the review of research performance,” in Advances in bibliometric analysis: research performance assessment and science mapping. Editors Blockmans W Engwall L. Weaire D. (London, UK: Portland Press), 17–28.

[B57] van Raan A. F. J. (2020). Urban scaling, geography, centrality: relation with local government structures. PLoS One 15 (9), e0238418e0238418. 10.1371/journal.pone.0238418 32886689 PMC7473566

[B58] Waltman L. (2017). A review of the literature on citation impact indicators. ArXiv. Available at: https://arxiv.org/abs/1507.02099 (Accessed September 18, 2017).

